# Case Report: Management of Polycaprolactone‐Based Dermal Filler–Induced Vascular Occlusion

**DOI:** 10.1111/jocd.16779

**Published:** 2025-01-06

**Authors:** Shirin Zaresharifi, Mehdi Gheisari, Hamideh Mohammadzadeh, Hojjat Layegh, Maliheh Amani

**Affiliations:** ^1^ Skin Research Center Shahid Beheshti University of Medical Sciences Tehran Iran; ^2^ Department of Dermatology, Loghman‐Hakim Hospital, School of Medicine Shahid Beheshti University of Medical Sciences Tehran Iran; ^3^ Department of Dermatology, Faculty of Medicine Gonabad University of Medical Sciences Gonabad Iran; ^4^ Department of Plastic and Reconstructive Surgery Shahid Beheshti University of Medical Sciences Tehran Iran

## 1 Case Presentation

**FIGURE 1:**
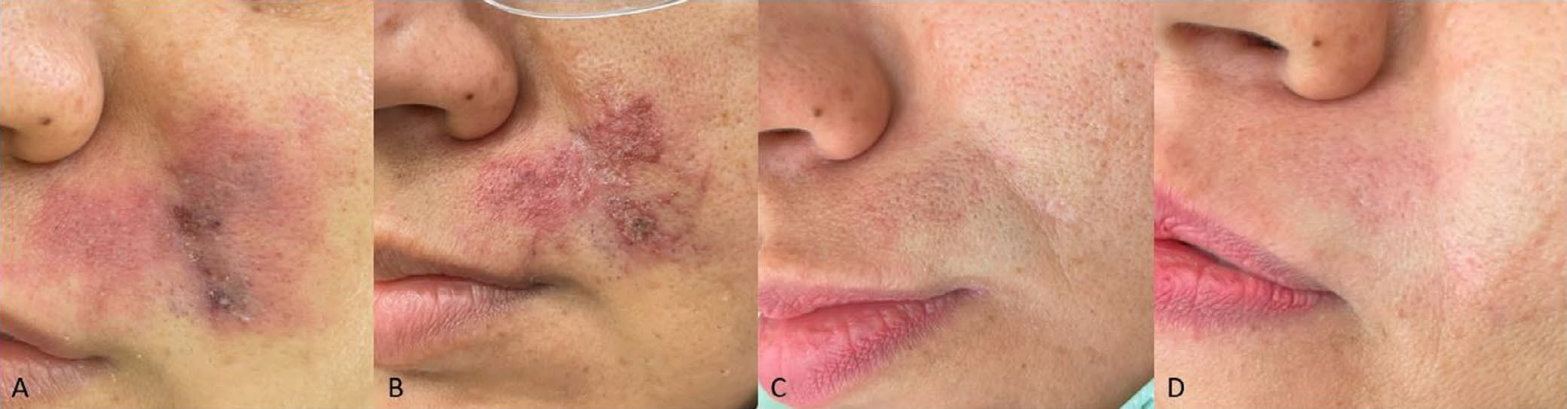
Sequential clinical photographs showing the progression of treatment following polycaprolactone‐induced vascular occlusion in a 35‐year‐old female. (A) Taken on Day 3 post‐injection, the image shows marked erythema and dusky discoloration, indicative of early ischemic changes. (B) By Week 1, reticular areas and violaceous discoloration with crust formation are visible in the affected area, suggesting ongoing ischemia but some degree of healing, this picture is before initiating CO_2_ and platelet‐rich plasma. (C) After 4 weeks, small areas of scarring are observed. (D) After 5 months, complete resolution of erythema and discoloration is noted, with minimal residual scarring and excellent cosmetic recovery after fractional CO_2_.

A 42‐year‐old female presented to the clinic 3 days after receiving polycaprolactone (PCL)‐based dermal filler injections in the hypodermal layer of nasolabial folds by the use of a needle. She had received 0.5 cc of PCL‐based filler in each nasolabial fold under local anesthesia. She did not mention any pain during the injection. However, she noticed erythema and pain in the left nasolabial fold 1 day after the procedure, which she did not find significant and did not seek medical care for it; however, as the symptoms progressively worsened despite initial conservative management with over‐the‐counter analgesics she visited our clinic (Figure 1).

Her medical history was unremarkable, and she did not report any prior history of dermal filler injection in this area. The filler injection had been performed without immediate adverse events. Physical examination revealed erythema, tenderness, and moderate edema along the left nasolabial fold; there were also areas of dusky erythema and pustule formation indicating impending necrosis in the area. Her physical examination was otherwise unremarkable.

## 2 Treatment Initiated

Based on the clinical presentation, vascular occlusion was promptly diagnosed. A capillary refill test was conducted, revealing a delayed refill time of 4 s, slower than that of the contralateral tissue. The treatment protocol was initiated immediately, following the sequence outlined below:

1. Hyaluronidase injection: Although PCL fillers are not hyaluronic acid (HA)–based, hyaluronidase was administered to potentially relieve the compression caused by the filler and enhance vascular perfusion. A total of 500 units of hyaluronidase were injected into the hypodermis in the affected regions every hour for three consecutive hours; this procedure was repeated over the next 2 days.
2. Triamcinolone injection: The patient was administered 15 mg/mL of triamcinolone acetonide, injected intradermally 1 cm apart, with 0.1 cc injected at each point in the affected regions.
3. Low‐molecular‐weight heparin (LMWH): The patient was prescribed enoxaparin for five consecutive days to prevent vascular thrombus formation. This was administered subcutaneously in the periumbilical area to reduce the risk of further vascular complications.
4. Antibiotics: To prevent secondary bacterial infection, the patient was given clindamycin 300 mg every 12 h for 1 week.

Following the initial phase of occlusion management, the capillary refill test was reassessed, showing an improved refill time of 2 s. Procedures to address scarring and dyspigmentation were promptly initiated as follows:

1. Platelet‐rich plasma (PRP): On the fourth day, after the initial edema subsided, PRP therapy was initiated to promote healing and tissue regeneration. A second PRP session was performed 1 week later.
2. CO_2_ fractional laser therapy: Four weeks after the onset of symptoms, CO_2_ fractional laser therapy was initiated to address any potential post‐inflammatory hyperpigmentation or scarring. The patient received treatments every 4 weeks, for four sessions.

After 5 months of follow‐up, the patient healed with minimal scarring and an excellent cosmetic outcome.

## 3 Discussion

PCL‐based fillers are generally considered safe for soft tissue augmentation due to their biocompatibility and longevity. However, as demonstrated in this case, vascular occlusion remains a significant risk associated with all dermal fillers, particularly in areas such as the nasolabial folds, where major arteries are present [1]. Vascular occlusion can occur due to direct injection into a vessel, or external compression of nearby vasculature by the filler material and inflammation. This can lead to ischemia and, if untreated, tissue necrosis and significant scarring [2].

Anatomical factors play a pivotal role in the occurrence of such complications. The facial artery, which arises from the external carotid artery, follows a tortuous course across the face, making it highly susceptible to inadvertent cannulation or compression. In the nasolabial fold, the facial artery and its branches, particularly the superior labial and angular arteries, lie close to typical filler injection planes within the dermis or hypodermis. The variable depth and tortuosity of the facial artery further complicate this risk. While in most individuals, it runs deeply in the lower two‐thirds of the nasolabial fold before becoming more superficial in the upper one‐third, in some patients, it may course superficially, lying within millimeters of the dermis even in the lower part, or it may be situated deeply at the alar base. This variability can lead to unpredictable outcomes, even when standard injection techniques are employed [3].

Furthermore, areas such as the glabella and the periorbital regions are considered “high‐risk zones” due to the presence of critical anastomoses between the facial and ophthalmic arterial systems. Accidental intravascular injection in these regions can result in severe complications, including blindness or cerebrovascular events. These anatomical risks are further exacerbated by the use of sharp needles, rapid filler injections, or excessive injection pressure, particularly when using non‐HA fillers [4].

The use of blunt cannulas, aspiration prior to injection, and slow, controlled filler deposition significantly mitigates vascular complications. Advanced imaging techniques, such as Doppler ultrasound, further enhance safety by enabling real‐time visualization of vascular structures, particularly in high‐risk areas or regions with common anatomical variations [5].

Several case reports and studies have described successful management strategies for vascular occlusion caused by non‐HA fillers, including PCL. This case adds to the limited literature on PCL‐induced vascular complications and highlights the importance of early recognition and intervention to prevent serious outcomes [6].

### 3.1 Treatment Options Discussed in the Literature

Before initiating treatment, it is advisable to evaluate vascular flow using the capillary refill time. This can be done by applying moderate pressure to the ischemic area and comparing the results to those of the adjacent normal tissue. Additionally, vascular flow can be assessed more accurately through ultrasound imaging when available [7].

Treatment strategies can be categorized into two main groups: immediate interventions addressing vascular occlusion, hypoxemia, and tissue necrosis, and subsequent treatments focused on minimizing complications such as scarring and dyspigmentation.

### 3.2 Immediate Treatments for Vascular Occlusion

These interventions focus on restoring blood flow, alleviating hypoxemia, and preventing further tissue damage.

Hyaluronidase: Although primarily used for HA fillers, hyaluronidase was used in this case to improve vascular perfusion by potentially reducing tissue compression by degrading carrier gel and breaking down surrounding tissues, and therefore alleviating compression of the injected area. The literature strongly supports its use in cases of vascular occlusion, even with non‐hyaluronic fillers [8, 9].

Corticosteroids: Corticosteroids, both through local injection and systemic administration, play a critical role in managing the inflammation caused by non‐HA filler vascular occlusion. By rapidly reducing inflammation, they prevent further tissue damage, improve circulation, promote healing, and reduce subsequent scarring [10].

Anticoagulants: Both heparin, including LMWH, for example, enoxaparin, and aspirin can play critical roles in the management of vascular occlusion caused by non‐HA dermal fillers. LMWH, as an anticoagulant, prevents clot formation and improves blood flow in compromised vessels, while aspirin, as an antiplatelet agent, prevents platelet aggregation and reduces the risk of thrombus formation. These agents, especially when used in conjunction with other therapies, can mitigate the risks of tissue ischemia and necrosis following vascular occlusion [11, 12].

Nitroglycerin paste: Although not generally recommended in vascular compromise caused by HA fillers, in cases involving non‐HA fillers, nitroglycerin paste is often recommended as part of the initial treatment protocol for suspected vascular occlusion, by inducing vasodilation and promoting reperfusion in the affected ischemic area [6].

5‐Fluorouracil (5‐FU): Another notable case report by Guo et al. described the use of 5‐FU as part of a treatment regimen for facial artery embolization caused by a PCL‐based filler. This report represents one of the few documented cases of vascular occlusion due to PCL fillers, further emphasizing the importance of early, multi‐modal intervention in these cases. In Zhuang's report, 5‐FU was used alongside corticosteroids and anticoagulants to reduce ischemia and promote tissue recovery. This treatment approach, including the use of 5‐FU, highlights the growing recognition of adjunctive therapies beyond standard vasodilators and anticoagulants [13].

Hyperbaric oxygen therapy (HBOT): HBOT is also recommended as an adjunctive treatment for vascular occlusion by non‐HA fillers. HBOT increases oxygen delivery to ischemic tissues by promoting oxygen saturation in plasma and tissues. This can aid in reducing tissue ischemia and preventing necrosis, particularly in cases where oxygen deprivation is severe. The literature suggests that early administration of HBOT improves tissue survival by increasing perfusion and facilitating the repair of damaged skin and subcutaneous tissue. It is particularly useful when other interventions are insufficient to restore full vascular function [14].

Oral vasodilators: While oral vasodilators like PDE5 inhibitors and prostaglandin E1 are suggested for vascular occlusion, evidence for their effectiveness is limited. Sildenafil, although commonly recommended, lacks direct studies supporting its efficacy and may cause adverse effects, including blood pressure drops, especially with oral nitrates [7].

### 3.3 Treatments to Mitigate Scarring and Dyspigmentation

These therapies aim to reduce the long‐term aesthetic and functional consequences of vascular occlusion.

PRP: PRP is increasingly used as an adjunct therapy in the management of filler complications due to its regenerative properties and its enhancement of wound healing [15].

CO_2_ laser: Fractional CO_2_ laser therapy is a well‐established modality for managing post‐inflammatory pigmentation and reducing scar formation following tissue damage from ischemia [16].

The literature emphasizes early recognition and intervention as key factors in preventing irreversible tissue damage. In cases like this, immediate treatment with hyaluronidase, corticosteroids, and other supportive measures is crucial. Adjunctive treatments such as PRP and laser therapy can further enhance healing and minimize long‐term cosmetic sequelae.

Reported cases of vascular occlusion due to PCL fillers are rare, making each documented case particularly valuable for practitioners. This case, alongside the few other case reports on this subject, serves as a critical reference point for managing such complications. Articles in this area are limited, but an animal‐based study by Khan et al. demonstrated that the combination of heparin and nitroglycerin significantly improved blood flow and reduced tissue damage in an animal model of PCL filler‐induced embolism. While the results are promising, further human studies are needed to confirm its clinical utility [6]. These cases underscore the importance of a multidisciplinary approach in managing vascular occlusion, involving corticosteroids, vasodilators, anticoagulation, and adjunctive therapies such as 5‐FU and laser treatments.

## 4 Conclusion

This case highlights the importance of early diagnosis and prompt treatment of vascular occlusion after PCL‐based dermal filler injections. A combination of corticosteroids, hyaluronidase, and LMWH, along with supportive therapies like CO_2_ laser and PRP, resulted in the successful resolution of symptoms and prevention of long‐term damage and permanent scarring. This case underscores the need for practitioners to be aware of the potential risks and treatment protocols for vascular occlusion following non‐HA filler injections.

## Author Contributions

M.A. and H.M. performed the research. S.Z. and H.L. wrote the paper. M.G. revised and supervised the manuscript.

## Consent

We confirm that written patient consent has been signed and collected from each patient, in accordance with the journal's patient consent policy. We will retain the original written consent forms and provide them to the publisher if requested.

## Conflicts of Interest

The authors declare no conflicts of interest.

## Data Availability

The data that support the findings of this study are available from the corresponding author upon reasonable request.
